# Tubercular Meningitis

**Published:** 1894-01

**Authors:** W. H. Mattsen


### TUBERCULAR MENINGITIS.

By W. H. Mattsen, V.M.D.

One of our M. D.'s called my attention to a 6-months'-old Jersey calf he was raising, which had, to within a week of my seeing it, seemed perfectly healthy. It was in as good condition as calves of that age generally are, and seemed to be thriving nicely.

*Symptoms and History.*—The doctor said he was in the field looking at this calf one afternoon, when very suddenly it fell to its side, was unable to get up without help, and if let go would fall again ; its eyes would roll back ; its head turned to one side (to the right), and it lay on its left side; it eat some, seemed hungry. He examined it the best he could and made his diagnosis ; finally he called my attention to it as being an interesting case, so I went to see it; found it on its left side with its back curved. I turned it over on the right side, when it immediately had a spasm; the neck was then bowed up and would not straighten. I turned it back again, when it was easier. I examined right lung and found all the sounds of tuberculosis; the left lung I did not listen to. I diagnosed him tuberculosis with, I thought, tubercles in the brain, or tubercular meningitis. That evening it died. The next day I held an autopsy. I found left lung solid, right lung two-thirds filled up; pleura very much thickened and covered with tubercles, some as large as a walnut; pericardium very thick, also covered with tubercles; the pleura had adhered to the ribs in many places; the liver and omentum were free from tubercles, as far as we could see with the eye. I took out the brain, but could not find anything, so I gave it to Dr. Formad of the University to examine under the microscope. To-day he wrote me that he found several tubercles on the blood vessel walls of the pia-mater and distinct lesions of tubercular meningitis, the brain matter itself being free from tubercles.

This was a peculiar case, as this calf had never shown any signs of *any* trouble whatever ; had never even been heard to cough. Diagnosis of dislocated vertebra had been made before I saw the case.

